# μ-Pyrazine-bis­[tetra­aqua­cadmium(II)] μ-pyrazine-bis­[tetra­acetato­cadmium(II)]

**DOI:** 10.1107/S1600536808010994

**Published:** 2008-04-30

**Authors:** Hoong-Kun Fun, Suchada Chantrapromma, Farzin Marandi

**Affiliations:** a X-ray Crystallography Unit, School of Physics, Universiti Sains Malaysia, 11800 USM, Penang, Malaysia; b Department of Chemistry, Faculty of Science, Prince of Songkla University, Hat-Yai, Songkhla 90112, Thailand; c Department of Science, Payame Noor University, Zanjan, Iran

## Abstract

In the title dinuclear ionic complex, [Cd_2_(C_4_H_4_N_2_)(H_2_O)_8_][Cd_2_(CH_3_CO_2_)_8_(C_4_H_4_N_2_)], the cation and anion are disordered equally over a site with symmetry *mmm*. The Cd^II^ ions and the N atoms of the bridging pyrazine ligand lie on the inter­section of two crystallographic mirror planes. The C atoms of the bridging pyrazine ligand lie on one of these mirror planes, and the acetate groups and water mol­ecules lie across the inter­secting mirror planes. Each Cd^II^ atom in the cation is five-coordinated by four O atoms from four water mol­ecules and one N atom from the bridging pyrazine ligand, whereas each Cd^II^ in the anion is nine-coordinated by four pairs of O atoms from the bidentate acetate ligands and one N atom from the bridging pyrazine ligand. In the crystal structure, each anion is surrounded by eight nearest-neighbour cations and *vice versa*. The crystal structure is stabilized by ionic inter­actions as well as by C—H⋯O inter­actions.

## Related literature

For bond-length data, see: Allen *et al.* (1987 ▶). For Cd^II^ coord­ination chemistry, applications and related structures, see: Filipović *et al.* (2008 ▶); Inoue *et al.* (2000 ▶); Pons *et al.* (2007 ▶); Xia *et al.* (2004 ▶). 
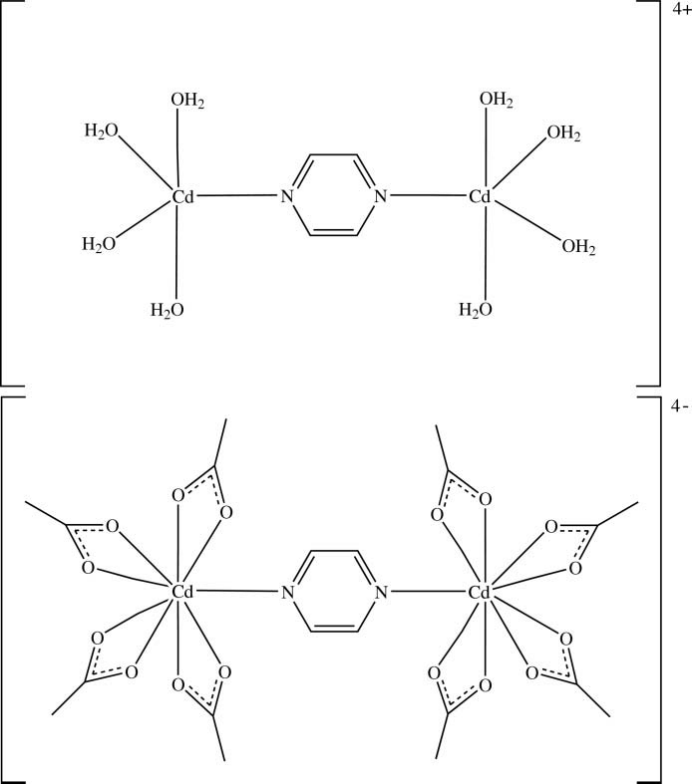


## Experimental

### 

#### Crystal data

[Cd_2_(C_4_H_4_N_2_)(H_2_O)_8_][Cd_2_(C_2_H_3_O_2_)_8_(C_4_H_4_N_2_)]
*M* *_r_* = 1226.26
Tetragonal, 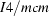

*a* = 16.7103 (4) Å
*c* = 7.3533 (2) Å
*V* = 2053.29 (9) Å^3^
*Z* = 2
Mo *K*α radiation
μ = 2.13 mm^−1^
*T* = 100.0 (1) K
0.58 × 0.08 × 0.05 mm

#### Data collection

Bruker SMART APEXII CCD area-detector diffractometer
Absorption correction: multi-scan (*SADABS*; Bruker, 2005 ▶) *T*_min_ = 0.371, *T*_max_ = 0.895
12617 measured reflections
1246 independent reflections
1145 reflections with *I* > 2σ(*I*)
*R*_int_ = 0.037

#### Refinement

*R*[*F* ^2^ > 2σ(*F* ^2^)] = 0.016
*wR*(*F* ^2^) = 0.034
*S* = 1.06
1246 reflections
73 parameters
2 restraints
H atoms treated by a mixture of independent and constrained refinement
Δρ_max_ = 0.59 e Å^−3^
Δρ_min_ = −0.44 e Å^−3^

### 

Data collection: *APEX2* (Bruker, 2005 ▶); cell refinement: *APEX2*; data reduction: *SAINT* (Bruker, 2005 ▶); program(s) used to solve structure: *SHELXTL* (Sheldrick, 2008 ▶); program(s) used to refine structure: *SHELXTL*; molecular graphics: *SHELXTL*; software used to prepare material for publication: *SHELXTL* and *PLATON* (Spek, 2003 ▶).

## Supplementary Material

Crystal structure: contains datablocks global, I. DOI: 10.1107/S1600536808010994/ci2576sup1.cif

Structure factors: contains datablocks I. DOI: 10.1107/S1600536808010994/ci2576Isup2.hkl

Additional supplementary materials:  crystallographic information; 3D view; checkCIF report

## Figures and Tables

**Table 1:** Hydrogen-bond geometry (Å, °)

*D*—H⋯*A*	*D*—H	H⋯*A*	*D*⋯*A*	*D*—H⋯*A*
C1—H1*A*⋯O2	0.96	2.47	3.0405 (18)	118
C1—H1*A*⋯O1*W*^i^	0.96	2.56	3.2700 (17)	131
C1—H1*A*⋯O2^ii^	0.96	2.47	3.0405 (18)	118
C1—H1*A*⋯O1*W*^iii^	0.96	2.56	3.2700 (17)	131

Symmetry codes: (i) 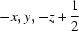; (ii) 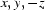; (iii) 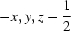.

**Fig. 1.**
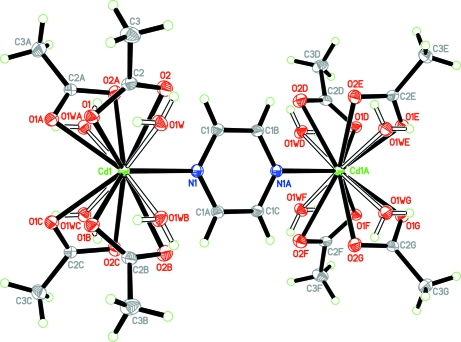
The molecular structure of the title compound, showing 50% probability displacement ellipsoids and the atomic numbering. The cations and anions occupy the same site with equal occupancy. Symmetry codes for the Cd1A ion: (A) -x, 1 - y, -z. Symmetry codes for aqua and acetate atoms: (A) x, y, -z; (B) 1/2 - y, 1/2 - x, z; (C) 1/2 - y, 1/2 - x, -z; (D) y - 1/2, 1/2 + x, -z; (E) y - 1/2, 1/2 + x, z; (F) -x, 1 - y, -z; (G) -x, 1 - y, z. Symmetry codes for ring C atoms: (A) 1/2 - y, 1/2 - x, z; (B) y - 1/2, 1/2 + x, -z; (C) -x, 1 - y, -z. Symmetry code for the N1 atom: (A) -x, 1 - y, -z.

**Fig. 2.**
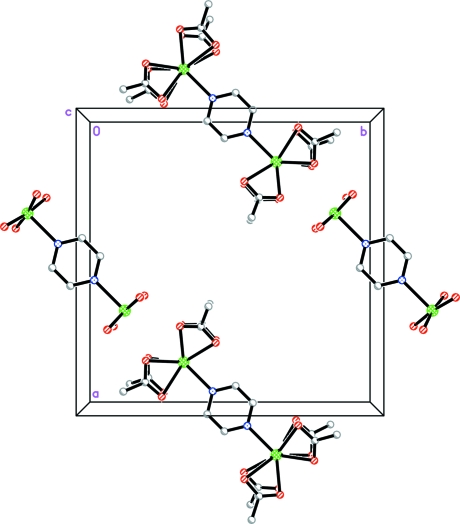
The crystal packing of the title compound, viewed along the c axis, showing a layer of the cations and anions.

**Fig. 3.**
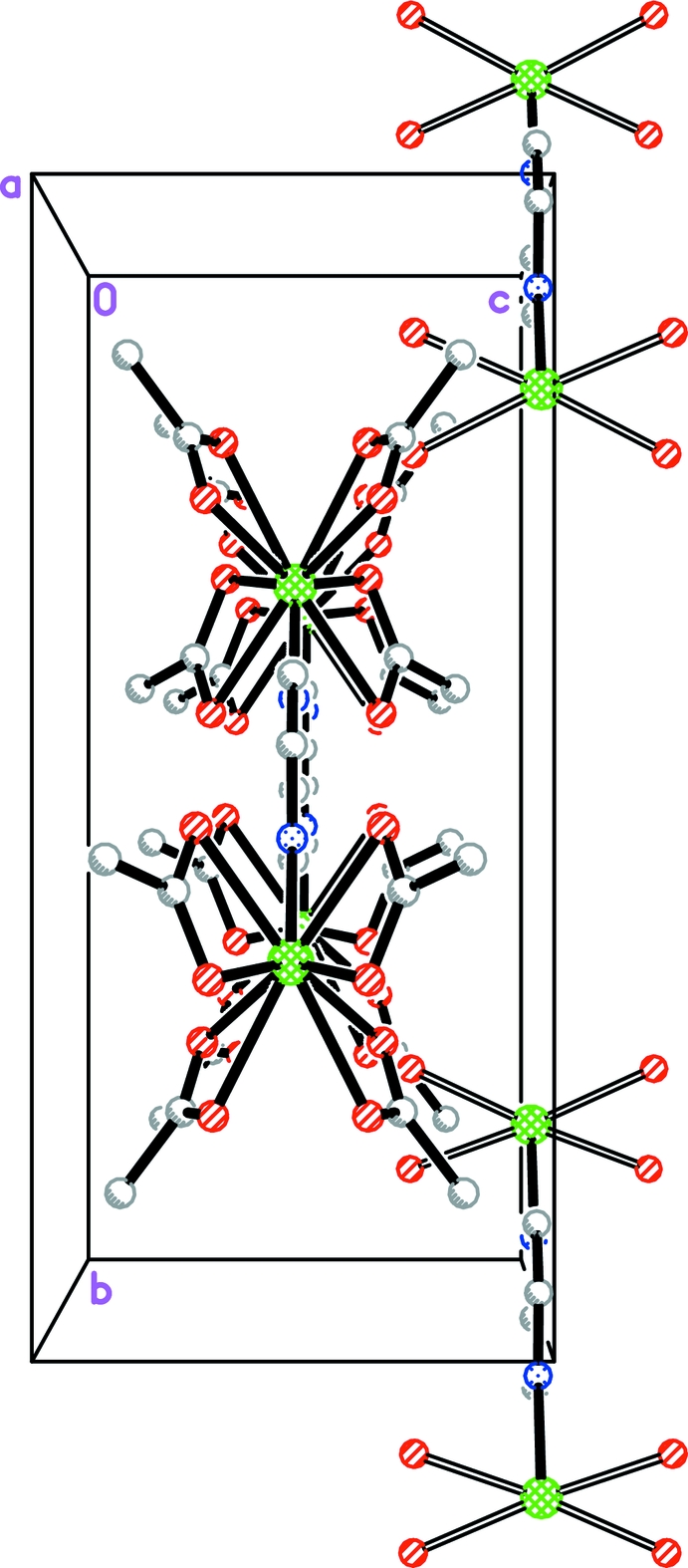
The crystal packing of the title compound, viewed along the a axis, showing the same layer of cations and anions in Fig. 2.

## supplementary crystallographic information

### Comment

The investigations of coordination compounds between cadmium(II) and O and N donors atoms have attracted much attention due to their potential application in a number of areas (Inoue *et al.*, 2000; Pons *et al.*, 2007; Xia *et al.*, 2004), including cytotoxic activities (Filipović *et al.*, 2008). We report herein, the synthesis and crystal structure of the title compound which exhibits a mixed coordination for Cd^II^ atom i.e nine- and five-coordination mode for Cd^II^, which is a rare case.

In the title compound, both [Cd_2_(H_2_O)_8_(C_4_H_4_N_2_)]^4+^ cations and [Cd_2_(CH_3_CO_2_)_8_(C_4_H_4_N_2_)]^4-^ anions lie on the site symmetry *mmm*. The Cd^II^ and the N atoms of the bridging pyrazine ligand lie on the intersection of two crystallographic mirror planes, one perpenidicular to the *c* axis (*z* = 0) and the other parallel to the *c* axis and passing through the mid points of the *a* and *b* axis. The C atoms of the bridging pyrazine ligand lie on the *z* = 0 mirror plane, and the acetate groups and water molecules lie across the intersecting mirror planes. There are two molecules of the title complex in the unit cell.

In the structure, the cation contains two Cd^II^ ions, eight water molecules and one bridging pyrazine ligand whereas the anion contains two Cd^II^ ions, eight acetate and one bridging pyrazine ligands. Each of the Cd^II^ in the cation is five-coordinated with four O atoms from four water molecules and one N atom from a bridging pyrazine ligand, whereas each Cd^II^ in the anion is nine-coordinated with four pairs of chelate O atoms from the bidentate acetate ligands and one N atom from the bridging pyrazine ligand. The Cd—*O*(acetate) bond distances are 2.3458 (14) and 2.5200 (14) Å, and the Cd—O(water) and Cd—N distances are 2.2783 (15) Å and 2.3470 (15) Å, respectively. The O(water)—Cd—O(water) bond angles lie in the range 64.15 (8)°-174.10 (7)°, whereas, the *O*(acetate)—Cd—*O*(acetate) bond angles are in the range 53.57 (7)°-158.87 (6)°. The N—Cd—O(water) angle is 92.95 (3)° and *N*—Cd—*O*(acetate) angles are 132.77 (3)° and 79.43 (3)°, respectively. The geometric parameters are comparable to those reported for other Cd—O and Cd—N donor complexes (Inoue *et al.*, 2000; Pons *et al.*, 2007; Xia *et al.*, 2004).

In the crystal packing (Fig. 2 and Fig.3), each anion is surrounded by eight nearest neighbour cations and *vice*-*versa*. The crystal structure is stablized by ionic interactions as well as by weak C—H···O interactions (Table 1).

### Experimental

The title compound was synthesized by mixing Cd(CH_3_COO)_2_ and pyrazine with a 2:1 molar ratio in a hot methanol-water (2:1 *v*/*v*) solution and stirred for 10 min at room temperature. The solution was then left at ambient temperature to allow the solvent to slowly evaporate. Colourless crystals of the title compound suitable for X-ray structure determination were obtained after a few weeks.

### Refinement

Water H atoms were located in a difference map and refined isotropically, with a O—H distance restraint of 0.800 (1) Å. C-bound H atoms were positioned geometrically and allowed to ride on their parent atoms, with C—H = 0.96 Å. The *U*_iso_ value of H1A was constrained to 1.2*U*_eq_(C1) and for other H atoms the *U*_iso_ values were refined. A rotating group model was used for the methyl group.

### Crystal data

**Table:**

[Cd_2_(C_4_H_4_N_2_)(H_2_O)_8_][Cd_2_(C_2_H_3_O_2_)_8_(C_4_H_4_N_2_)]	*D*_x_ = 1.983 Mg m^−^^3^
*M**_r_* = 1226.26	Mo *K*α radiation, λ = 0.71073 Å
Tetragonal, *I*4/*m**c**m*	Cell parameters from 1246 reflections
Hall symbol: -I 4 2c	θ = 1.7–35.0°
*a* = 16.7103 (4) Å	µ = 2.13 mm^−^^1^
*c* = 7.3533 (2) Å	*T* = 100 K
*V* = 2053.29 (9) Å^3^	Needle, colourless
*Z* = 2	0.58 × 0.08 × 0.05 mm
*F*(000) = 1208	

### Data collection

**Table:**

Bruker SMART APEXII CCD area-detector diffractometer	1246 independent reflections
Radiation source: fine-focus sealed tube	1145 reflections with *I* > 2σ(*I*)
graphite	*R*_int_ = 0.037
Detector resolution: 8.33 pixels mm^-1^	θ_max_ = 35.0°, θ_min_ = 1.7°
ω scans	*h* = −26→26
Absorption correction: multi-scan (*SADABS*; Bruker, 2005)	*k* = −26→26
*T*_min_ = 0.371, *T*_max_ = 0.895	*l* = −11→11
12617 measured reflections	

### Refinement

**Table:**

Refinement on *F*^2^	Primary atom site location: structure-invariant direct methods
Least-squares matrix: full	Secondary atom site location: difference Fourier map
*R*[*F*^2^ > 2σ(*F*^2^)] = 0.016	Hydrogen site location: inferred from neighbouring sites
*wR*(*F*^2^) = 0.034	H atoms treated by a mixture of independent and constrained refinement
*S* = 1.06	*w* = 1/[σ^2^(*F*_o_^2^) + (0.0089*P*)^2^ + 1.5621*P*] where *P* = (*F*_o_^2^ + 2*F*_c_^2^)/3
1246 reflections	(Δ/σ)_max_ = 0.002
73 parameters	Δρ_max_ = 0.59 e Å^−^^3^
2 restraints	Δρ_min_ = −0.44 e Å^−^^3^

### Special details

**Table:**

Experimental. The data was collected with the Oxford Cyrosystem Cobra low-temperature attachment.
Geometry. All e.s.d.'s (except the e.s.d. in the dihedral angle between two l.s. planes) are estimated using the full covariance matrix. The cell e.s.d.'s are taken into account individually in the estimation of e.s.d.'s in distances, angles and torsion angles; correlations between e.s.d.'s in cell parameters are only used when they are defined by crystal symmetry. An approximate (isotropic) treatment of cell e.s.d.'s is used for estimating e.s.d.'s involving l.s. planes.
Refinement. Refinement of *F*^2^ against ALL reflections. The weighted *R*-factor *wR* and goodness of fit *S* are based on *F*^2^, conventional *R*-factors *R* are based on *F*, with *F* set to zero for negative *F*^2^. The threshold expression of *F*^2^ > σ(*F*^2^) is used only for calculating *R*-factors(gt) *etc*. and is not relevant to the choice of reflections for refinement. *R*-factors based on *F*^2^ are statistically about twice as large as those based on *F*, and *R*- factors based on ALL data will be even larger.

### Fractional atomic coordinates and isotropic or equivalent isotropic displacement parameters (Å^2^)

**Table:**

	*x*	*y*	*z*	*U*_iso_*/*U*_eq_	Occ. (<1)
Cd1	0.158218 (5)	0.341782 (5)	0.0000	0.01214 (4)	
O1	0.16811 (7)	0.21686 (8)	0.1438 (2)	0.0156 (2)	0.50
O2	0.04830 (8)	0.27097 (9)	0.1708 (2)	0.0183 (3)	0.50
C2	0.09773 (11)	0.21621 (11)	0.2069 (3)	0.0144 (3)	0.50
C3	0.07138 (12)	0.14714 (12)	0.3238 (3)	0.0212 (4)	0.50
H3A	0.0344	0.1659	0.4142	0.036 (9)*	0.50
H3B	0.1172	0.1240	0.3825	0.038 (8)*	0.50
H3C	0.0458	0.1075	0.2493	0.031 (8)*	0.50
O1W	0.11198 (8)	0.28563 (8)	0.2621 (2)	0.0170 (3)	0.50
H1W1	0.0654 (4)	0.2746 (17)	0.262 (4)	0.020 (7)*	0.50
H2W1	0.1447 (16)	0.2523 (16)	0.287 (5)	0.055 (11)*	0.50
N1	0.05890 (6)	0.44110 (6)	0.0000	0.0131 (3)	
C1	−0.01905 (7)	0.42237 (7)	0.0000	0.0144 (2)	
H1A	−0.0354	0.3673	0.0000	0.017*	

### Atomic displacement parameters (Å^2^)

**Table:**

	*U*^11^	*U*^22^	*U*^33^	*U*^12^	*U*^13^	*U*^23^
Cd1	0.00955 (4)	0.00955 (4)	0.01731 (7)	0.00080 (4)	0.000	0.000
O1	0.0119 (5)	0.0133 (5)	0.0216 (7)	0.0003 (4)	0.0008 (5)	0.0015 (5)
O2	0.0137 (6)	0.0151 (6)	0.0260 (8)	0.0028 (5)	0.0007 (6)	0.0037 (5)
C2	0.0131 (7)	0.0118 (7)	0.0181 (8)	−0.0016 (5)	−0.0025 (6)	0.0004 (6)
C3	0.0189 (9)	0.0195 (9)	0.0251 (10)	−0.0014 (7)	0.0006 (7)	0.0074 (8)
O1W	0.0121 (5)	0.0156 (6)	0.0233 (7)	0.0022 (5)	0.0023 (5)	0.0030 (5)
N1	0.0119 (4)	0.0119 (4)	0.0155 (7)	0.0010 (5)	0.000	0.000
C1	0.0122 (5)	0.0113 (5)	0.0196 (6)	0.0004 (4)	0.000	0.000

### Geometric parameters (Å, °)

**Table:**

Cd1—O1W	2.2782 (15)	O1—C2	1.264 (2)
Cd1—O1W^i^	2.2783 (15)	O2—C2	1.261 (2)
Cd1—O1W^ii^	2.2783 (15)	C2—C3	1.505 (3)
Cd1—O1W^iii^	2.2783 (15)	C3—H3A	0.96
Cd1—O1	2.3458 (14)	C3—H3B	0.96
Cd1—O1^i^	2.3458 (14)	C3—H3C	0.96
Cd1—O1^ii^	2.3458 (14)	O1W—H1W1	0.800 (1)
Cd1—O1^iii^	2.3458 (14)	O1W—H2W1	0.800 (1)
Cd1—N1	2.3470 (15)	N1—C1^i^	1.3396 (15)
Cd1—O2^i^	2.5200 (14)	N1—C1	1.3397 (15)
Cd1—O2^ii^	2.5200 (14)	C1—C1^iv^	1.384 (2)
Cd1—O2	2.5200 (14)	C1—H1A	0.96
			
O1W—Cd1—O1W^i^	64.15 (8)	O1^i^—Cd1—O2^ii^	81.84 (5)
O1W—Cd1—O1W^ii^	174.10 (7)	O1^ii^—Cd1—O2^ii^	53.77 (4)
O1W^i^—Cd1—O1W^ii^	115.52 (8)	O1^iii^—Cd1—O2^ii^	113.02 (5)
O1W—Cd1—O1W^iii^	115.51 (8)	N1—Cd1—O2^ii^	79.43 (3)
O1W^i^—Cd1—O1W^iii^	174.10 (7)	O2^i^—Cd1—O2^ii^	59.77 (8)
O1W^ii^—Cd1—O1W^iii^	64.15 (8)	O1—Cd1—O2	53.77 (4)
O1—Cd1—O1^i^	70.82 (6)	O1^i^—Cd1—O2	113.02 (5)
O1—Cd1—O1^ii^	94.46 (6)	O1^ii^—Cd1—O2	147.15 (4)
O1^i^—Cd1—O1^ii^	53.57 (7)	O1^iii^—Cd1—O2	81.84 (5)
O1—Cd1—O1^iii^	53.57 (7)	N1—Cd1—O2	79.43 (3)
O1^i^—Cd1—O1^iii^	94.46 (6)	O2^i^—Cd1—O2	115.86 (8)
O1^ii^—Cd1—O1^iii^	70.82 (6)	O2^ii^—Cd1—O2	158.87 (6)
O1W—Cd1—N1	92.95 (3)	C2—O1—Cd1	96.17 (11)
O1W^i^—Cd1—N1	92.95 (3)	C2—O2—Cd1	88.18 (11)
O1W^ii^—Cd1—N1	92.95 (3)	O2—C2—O1	121.73 (17)
O1W^iii^—Cd1—N1	92.95 (3)	O2—C2—C3	119.02 (16)
O1—Cd1—N1	132.77 (3)	O1—C2—C3	119.23 (16)
O1^i^—Cd1—N1	132.77 (3)	Cd1—O1W—H1W1	115 (2)
O1^ii^—Cd1—N1	132.77 (3)	Cd1—O1W—H2W1	104 (3)
O1^iii^—Cd1—N1	132.77 (3)	H1W1—O1W—H2W1	120 (3)
O1—Cd1—O2^i^	113.02 (5)	C1^i^—N1—C1	117.01 (15)
O1^i^—Cd1—O2^i^	53.77 (4)	C1^i^—N1—Cd1	121.49 (8)
O1^ii^—Cd1—O2^i^	81.84 (5)	C1—N1—Cd1	121.49 (8)
O1^iii^—Cd1—O2^i^	147.15 (4)	N1—C1—C1^iv^	121.50 (8)
N1—Cd1—O2^i^	79.43 (3)	N1—C1—H1A	120.0
O1—Cd1—O2^ii^	147.15 (4)	C1^iv^—C1—H1A	118.5
			
O1^i^—Cd1—O1—C2	137.93 (10)	O1W^iii^—Cd1—N1—C1^i^	−122.12 (4)
O1^ii^—Cd1—O1—C2	−173.19 (14)	O1—Cd1—N1—C1^i^	142.13 (5)
O1^iii^—Cd1—O1—C2	−111.03 (11)	O1^i^—Cd1—N1—C1^i^	37.87 (5)
N1—Cd1—O1—C2	6.81 (14)	O1^ii^—Cd1—N1—C1^i^	−37.87 (5)
O2^i^—Cd1—O1—C2	103.79 (12)	O1^iii^—Cd1—N1—C1^i^	−142.13 (5)
O2^ii^—Cd1—O1—C2	173.28 (11)	O2^i^—Cd1—N1—C1^i^	30.46 (4)
O2—Cd1—O1—C2	−2.25 (11)	O2^ii^—Cd1—N1—C1^i^	−30.46 (4)
O1—Cd1—O2—C2	2.24 (11)	O2—Cd1—N1—C1^i^	149.54 (4)
O1^i^—Cd1—O2—C2	−38.85 (13)	O1W—Cd1—N1—C1	−57.88 (4)
O1^ii^—Cd1—O2—C2	19.05 (17)	O1W^i^—Cd1—N1—C1	−122.12 (4)
O1^iii^—Cd1—O2—C2	52.55 (12)	O1W^ii^—Cd1—N1—C1	122.12 (4)
N1—Cd1—O2—C2	−171.01 (12)	O1W^iii^—Cd1—N1—C1	57.88 (4)
O2^i^—Cd1—O2—C2	−98.33 (12)	O1—Cd1—N1—C1	−37.87 (5)
O2^ii^—Cd1—O2—C2	−171.01 (12)	O1^i^—Cd1—N1—C1	−142.13 (5)
Cd1—O2—C2—O1	−3.94 (19)	O1^ii^—Cd1—N1—C1	142.13 (5)
Cd1—O2—C2—C3	177.83 (17)	O1^iii^—Cd1—N1—C1	37.87 (5)
Cd1—O1—C2—O2	4.3 (2)	O2^i^—Cd1—N1—C1	−149.54 (4)
Cd1—O1—C2—C3	−177.52 (16)	O2^ii^—Cd1—N1—C1	149.54 (4)
O1W—Cd1—N1—C1^i^	122.12 (4)	O2—Cd1—N1—C1	−30.46 (4)
O1W^i^—Cd1—N1—C1^i^	57.88 (4)	C1^i^—N1—C1—C1^iv^	0.0
O1W^ii^—Cd1—N1—C1^i^	−57.88 (4)	Cd1—N1—C1—C1^iv^	180.0

Symmetry codes: (i) −*y*+1/2, −*x*+1/2, *z*; (ii) −*y*+1/2, −*x*+1/2, −*z*; (iii) *x*, *y*, −*z*; (iv) *y*−1/2, *x*+1/2, −*z*.

### Hydrogen-bond geometry (Å, °)

**Table:**

*D*—H···*A*	*D*—H	H···*A*	*D*···*A*	*D*—H···*A*
C1—H1A···O2	0.96	2.47	3.0405 (18)	118
C1—H1A···O1W^v^	0.96	2.56	3.2700 (17)	131
C1—H1A···O2^vi^	0.96	2.47	3.0405 (18)	118
C1—H1A···O1W^vii^	0.96	2.56	3.2700 (17)	131

Symmetry codes:  (v) −*x*, *y*, −*z*+1/2; (vi) *x*, *y*, −*z*; (vii) −*x*, *y*, *z*−1/2.

## References

[bb1] Allen, F. H., Kennard, O., Watson, D. G., Brammer, L., Orpen, A. G. & Taylor, R. (1987). *J. Chem. Soc. Perkin Trans. 2*, pp. S1–S19.

[bb2] Bruker (2005). *APEX2*, *SAINT* and *SADABS* Bruker AXS Inc., Madison, Wisconsin, USA.

[bb3] Filipović, N. R., Bacchi, A., Lazić, M., Pelizzi, G., Radulović, S., Sladić, D., Todorović, T. R. & Andelković, K. K. (2008). *Inorg. Chem. Commun.* **11**, 47–50.

[bb4] Inoue, M. B., Muñoz, I. C., Inoue, M. & Fernando, Q. (2000). *Inorg. Chim. Acta*, **300–302**, 206–211.

[bb5] Pons, J., García-Antón, J., Jiménez, R., Solans, X., Font-Bardia, M. & Ros, J. (2007). *Inorg. Chem. Commun.* **10**, 1554–1556.

[bb6] Sheldrick, G. M. (2008). *Acta Cryst.* A**64**, 112–122. 10.1107/S0108767307043930 18156677

[bb7] Spek, A. L. (2003). *J. Appl. Cryst.* **36**, 7–13.

[bb8] Xia, S.-Q., Hu, S.-M., Dai, J.-C., Wu, X.-T., Fu, Z.-Y., Zhang, J.-J. & Du, W.-X. (2004). *Polyhedron*, **23**, 1003–1009.

